# Primary Care Practitioners' Approaches to Deprescribing Opioids for Older Adults With Chronic Pain: A Qualitative Analysis

**DOI:** 10.1111/jgs.70438

**Published:** 2026-04-08

**Authors:** Brianna X. Wang, Julia H. Lindenberg, Shoshana J. Herzig, Mara A. Schonberg, Timothy S. Anderson

**Affiliations:** ^1^ Division of General Medicine, Beth Israel Deaconess Medical Center Boston Massachusetts USA; ^2^ Harvard Medical School Boston Massachusetts USA; ^3^ Division of General Internal Medicine University of Pittsburgh Pittsburgh Pennsylvania USA; ^4^ Center for Pharmaceutical Policy and Prescribing, Department of Medicine University of Pittsburgh Pittsburgh Pennsylvania USA; ^5^ Center for Healthcare Evaluation, Research, and Promotion, Veterans Affairs Pittsburgh Healthcare System Pittsburgh Pennsylvania USA

**Keywords:** chronic pain, deprescribing, older adults, opioids, primary care

## Abstract

**Background:**

Risks related to long‐term opioid therapy for chronic pain are high and may increase over time with aging. Deprescribing may be a beneficial intervention for older adults prescribed chronic opioids.

**Methods:**

Semi‐structured interviews with hypothetical clinical cases of older adults prescribed opioids for chronic pain: (1) low‐risk case: a patient prescribed low‐dose opioids without concerns; (2) moderate‐risk case: a patient with multimorbidity and concurrent benzodiazepine use prescribed moderate opioid doses; (3) high‐risk case: a patient prescribed high‐dose opioids with signs of an opioid use disorder (OUD). PCPs were asked, in an open‐ended fashion, to discuss whether they would initiate a deprescribing conversation, how they would approach deprescribing, and how they would approach a patient who declined recommendations to deprescribe.

**Participants and Setting:**

PCPs from a Massachusetts health system.

**Results:**

18 PCPs participated (56% female, 78% academic). More than half of PCPs would initiate a deprescribing conversation across the three cases. PCPs' approach to deprescribing and mitigating risks differed based on clinical risk. In low and moderate‐risk cases, PCPs emphasized a patient‐directed taper plan and education on opioid risks. In the high‐risk case, some PCPs were uncertain about initiating a deprescribing conversation due to concerns about the patient's mental health and the risk of illicit opioid use. Naloxone was infrequently recommended across the three cases, but in the high‐risk case, approximately half of PCPs suggested medications for OUD.

**Conclusions:**

PCPs reported that they would often initiate opioid deprescribing conversations with older adults, but were less confident in managing older adults with signs of OUD. PCPs require additional support to implement successful conversations on opioid deprescribing with older adults.

## Summary

- Key Points
- Risks related to long‐term opioid therapy are high and may increase over time with aging; thus, deprescribing may be a beneficial intervention for older adults prescribed chronic opioids.
- In this qualitative evaluation involving hypothetical clinical cases of older adults using chronic opioids, primary care practitioners widely varied in their approaches to discussing opioid deprescribing with older adults.
- While primary care practitioners reported that they would often initiate opioid deprescribing conversations with older adults on high dose opioids, they were less confident in managing older adults with signs of opioid use disorder.
- Why does this paper matter?
- This qualitative study demonstrates that primary care practitioners may benefit from additional training and clinical tools to equip them with the evidence and appropriate risk mitigation strategies to implement conversations on opioid prescribing and deprescribing with older adults.

## 1 Introduction

Over the past decade, increased attention to the risks of long‐term opioid use and the opioid overdose crisis has led to public health and health system efforts to encourage reduction of prescribed chronic opioids for many patients, with mixed results [1, 2, 3, 4, 5]. Recent guidelines discourage the use of opioids for chronic pain in most circumstances given increased risks of sedation, falls, respiratory depression, drug interactions, and overdose [6]. Despite this, many older adults are prescribed long‐term opioids for chronic pain and prior research suggests that both older adults and primary care practitioners (PCPs) view opioids as a potentially beneficial last‐line option when other therapies are ineffective or contraindicated due to medical comorbidities [7, 8]. As older adults make up a rising proportion of the population, many patients who may have begun taking chronic opioids decades ago now face higher risks related to opioids than they may have at younger ages, due to physiologic changes as well as increased risks related to comorbidities and polypharmacy. These risks include but are not limited to: dizziness, sedation, falls and fall‐related fractures, as well as cognitive impairment [9]. Furthermore, rates of opioid use disorder among older adults have tripled in recent years [10]. Due to the uncertain benefits and known risks of opioids in older adults, deprescribing may be a beneficial intervention. Deprescribing refers to the process in which patients and clinicians work carefully together to reduce medications for which existing or potential harms outweigh existing or potential benefits, in alignment with patient care goals [11]. In contrast, tapering may include patient‐centered deprescribing as well as forced tapering without patient agreement. Successful and safe opioid deprescribing can be complex processes that require ongoing communication with older adults and close long‐term follow‐up [12, 13, 14]. Prior qualitative studies have examined PCPs' views on barriers and facilitators to opioid deprescribing [15, 16], but less is known about clinicians' decision‐making on initiating deprescribing conversations with older adults and how clinicians approach the opioid tapering process.

Informing future educational and interventional efforts to support PCPs in safe opioid prescribing and deprescribing for older adults requires both an understanding of current clinical practices and the identification of perceived barriers to deprescribing conversations. In a prior qualitative study, we conducted semi‐structured interviews with PCPs and older adults prescribed opioids for chronic pain to identify experiences with and barriers to deprescribing conversations [8]. We found that older adults and PCPs had different views on opioid risks and that communication with patients about their opioids were challenging and infrequent. To further understand PCPs' clinical approaches to deprescribing, the current study presents the results of PCP discussions of how they would approach three hypothetical cases of older adults prescribed opioids for chronic pain with varying levels of risk of opioid‐related risks.

## 2 Methods

### 2.1 Setting, Participants, and Study Design

In this qualitative study, we conducted semi‐structured interviews at a large urban academic medical center in Massachusetts. This study followed the Consolidated Criteria for Reporting Qualitative Research (COREQ) reporting guideline for qualitative studies [17]. Interviews took place between September and December 2022. Potential PCP participants were identified from the staff of five primary care clinics: one academic general internal medicine clinic, one academic geriatrics clinic, and three community‐based primary care clinics. Attending clinicians and nurse practitioners were invited to participate via email. PCPs completed a brief questionnaire on their demographics and clinical practice prior to the interview.

### 2.2 Interview Guide

PCP interviews consisted of two parts conducted in a single one‐on‐one session: semi‐structured interviews and case‐based discussions. The current study focuses on the case‐based discussions. The results of the preceding semi‐structured interviews on experiences, barriers, and facilitators to prescribing and deprescribing opioids for older adults with chronic pain have been previously published [8]. The interview guide and cases were developed by the study team, which included three general internists and two members of the medical center's Opioid Care Committee, and were based on a review of opioid prescribing and broad deprescribing literature. Interview guides were reviewed with the US Deprescribing Research Network Stakeholder Engagement Council, which is comprised of stakeholders who represent patient, family, and various organizational perspectives [18].

The case‐based discussions consisted of three clinical cases of older adults prescribed opioids for chronic pain with ascending levels of opioid‐related risk (Box 1). For each case, participants were asked three open‐ended questions with targeted follow up questions: (1) whether and how they would approach discussing deprescribing with the patient, (2) how they would structure a taper and tapering goal if the patient was agreeable to reducing their opioid use, and (3) their approach to mitigating risk if the patient was not open to deprescribing (see complete interview guide in Table S1). Due to time constraints, not every PCP completed all cases.

BOX 1 **Clinical cases.**

Low‐Risk Case

A 76‐year‐old male patient has a history of chronic low back pain for which he has been taking oxycodone/acetaminophen 5 MG/325 MG twice a day for 10 years. During pain flares, which happen about once a year, he takes up to 2 pills twice a day. He tries to stretch to reduce the back pain and has completed physical therapy in the past. He has a history of well‐controlled hypertension and GERD. He is satisfied with this pain regimen and has not had any falls, memory concerns, or bothersome side effects attributed to the oxycodone.

Moderate‐Risk Case

An 84‐year‐old female patient has severe knee osteoarthritis for which she has been maintained on chronic opioids for 12 years. She takes oxycodone‐acetaminophen 10–325 mg, 2 tabs 3x/day. She also has a history of atrial fibrillation on anticoagulation, hypertension, and generalized anxiety disorder for which she takes lorazepam 0.5 mg BID. She walks with a cane and is not a candidate for knee replacement. She has not had any falls, reports no memory concerns, and lives with her husband. She reports her pain is adequately controlled.

High‐Risk Case

A 69‐year‐old male patient has a history of remote alcohol use disorder, acute pancreatitis resulting in chronic abdominal pain for which he has been maintained on chronic opioids for 20 years. He takes Oxycontin 80 mg BID and oxycodone 10 mg q4‐6 h for pain. He is on an opioid treatment agreement and has been followed regularly. After the recent death of his wife, he has been noted to miss regular scheduled appointments, call in early for refills, and his last toxicology screen demonstrated oxycodone but also fentanyl. He reports that due to escalating pain he is buying oxycodone tablets on the street. He has not had any overdoses.

### 2.3 Qualitative Analysis

Interviews were conducted virtually by a study clinician, with a research assistant present who took field notes. All sessions were audio‐recorded and de‐identified. Recordings were transcribed using NVivo transcription and analyzed using theNVivo software, v12 (QSR International), and were reviewed for accuracy. A qualitative descriptive approach with conventional content analysis was used [19]. Two investigators (JHL, TSA) reviewed the transcripts to develop a codebook and independently identified preliminary themes using an inductive coding approach. After the initial coding process, the full study team met to organize codes into larger categories to reflect major themes in the data. Disagreement about the meaning of themes or codes was discussed and resolved by consensus. Exemplary themes and quotes were produced from the transcripts. Participant responses were organized by question and clinical case. Responses were quantified on whether or not PCPs would initiate a deprescribing conversation (would initiate, unsure, would not initiate), what the taper goal was (dose reduction, taper off, not specified), and whether they would recommend pharmacotherapy treatments for risk mitigation (naloxone, medications for OUD (MOUD), not mentioned). Fisher's exact tests were used to test for differences in PCP responses, with statistical significance set at a 2‐sided α = 0.05. The study was determined to be exempt human subjects' research by the Beth Israel Deaconess Medical Center institutional review board.

## 3 Results

18 PCPs participated, of which 10 (56%) were female, 14 (78%) practiced in an academic clinic, and 10 (56%) had greater than 10 years of clinical experience. All PCPs prescribed chronic opioids, 15 (83%) had an X‐waiver to prescribe buprenorphine, and 4 (22%) reported currently prescribing buprenorphine for OUD (Table 1). Thirteen participants completed the low‐risk case, 18 completed the moderate‐risk case, and 16 completed the high‐risk case (Table S2).

**TABLE 1:** Participant characteristics.

Characteristic	No. (%)
Demographics	
Overall	18 (100)
Age, years	
30–39	7 (39)
40–49	5 (28)
50–59	4 (22)
60+	2 (11)
Female	10 (56)
Race	
Asian	5 (28)
White	12 (67)
Two or More Races	1 (6)
Hispanic or Latino Ethnicity	2 (11)

Practice characteristics	No. (%)
Career	
Physician	17 (94)
Nurse Practitioner	1 (6)
Location	
Academic	14 (78)
Community	4 (22)
Years as an attending	
< 5 years	4 (22)
5–9 years	4 (22)
≥ 10 years	10 (56)
Time Spent in Clinic	
< 25%	2 (11)
25%–50%	3 (17)
50%–75%	6 (33)
> 75%	7 (39)
Panel size	
< 500	7 (39)
500–1000	5 (28)
> 1000	6 (33)
No. patients prescribed opioids	
1–24	15 (83)
25–50	3 (17)
X‐Waiver	
Yes	15 (83)
No	3 (17)
Currently prescribing buprenorphine	
Yes	4 (22)
No	14 (78)

### 3.1 Initiation of Opioid Deprescribing Conversations

For the low‐risk case, approximately half of the PCPs reported that they would have a conversation about reducing opioid use; several would talk to the patient about the risks and benefits of opioids but not in the context of reducing, and two said they would not discuss opioids. For the moderate‐risk case, most would have a conversation about reducing opioids, some would have a conversation about risks but not tapering, and one would not discuss opioids. For the high‐risk case, approximately half would initiate a conversation on reducing opioids while the other half of PCPs would prioritize taking steps to address addiction risk and/or the mental health of the patient as a higher priority than immediately reducing their opioid dose (Figure S1A).

Clinical approaches to initiating a deprescribing conversation differed based on clinical risk with aging, benzodiazepine co‐prescribing, and mental health emerging as specific themes that inform deprescribing conversations (Table 2). PCPs reported framing a deprescribing conversation around opioid risks related to aging in both the low‐ and moderate‐risk cases. For example, one PCP stated they would frame the conversation to a patient around the effect of aging on multimorbidity and mobility “As you get older and start to accumulate more medical issues and limitations in your mobility, we might find ourselves in a situation where it no longer is so safe to keep you on this current regimen” while another PCP focused on the effects of aging on metabolism, stating “I would share that older folks metabolize opioids differently, are more susceptible to side effects, and then talk about her specific situation… so that she knows what my concerns are and what my motivation is”. PCPs who would not initiate a deprescribing conversation in the high‐risk case would prioritize mental health concerns and establish social support prior to deprescribing. One PCP expressed they “would call the urgent social worker to see them, start something for depression, connect him with an intensive outpatient program” (PCP 8). PCPs' urgency to initiate a deprescribing conversation also varied by risk level. PCPs did not articulate immediate concerns motivating deprescribing in the low‐risk case as one PCP stated, “he seems pretty stable and doesn't have any reasons to come off of it” (PCP15). PCPs reported an increased impetus to initiate a deprescribing conversation for the moderate‐risk case based on elevated risk for adverse side effects due to aging and concurrent use of opioids and benzodiazepines. Three PCPs stated they would focus the conversation on reducing benzodiazepine use as a higher priority than reducing opioid use, with one stating “My first approach would be to reduce or eliminate the lorazepam because I think the polypharmacy would be my biggest concern off the bat”. For the high‐risk case, all PCPs would address urgent concerns about patient safety and addiction risk.

**TABLE 2:** Clinical reasoning on approaching an opioid deprescribing conversation.

Theme	Exemplary quotes
Aging is a trigger to reassess risks in low‐ and moderate‐ risk cases.	“I would have a conversation about potential side effects. Do you find over the years that it has been less helpful than it was initially? In which case we should talk about something that works better.” (PCP 10, low‐risk) “As you get older and start to accumulate more medical issues and limitations in your mobility, we might find ourselves in a situation where it no longer is so safe to keep you on this current regimen.” (PCP 12, low‐risk) “I would share that older folks metabolize opioids differently, are more susceptible to side effects, and then talk about her specific situation… so that she knows what my concerns are and what my motivation is.” (PCP 17, moderate‐risk)
Urgency to initiate a deprescribing conversation varied by risk level.	“If I'm meeting him for the first time, I would absolutely address his opiates as a risk for the next 20 years of his life. I don't know that I would push him particularly hard because…he's pretty healthy and he's not suffering any side effects right now.” (PCP 3, low‐risk) “There's little benefit to continuing this level of opioid treatment. Higher doses are not helping. The fact that there is fentanyl in his urine toxicology puts him at extremely high risk for overdose death. And it's also a violation of our agreement” (PCP 7, high‐risk)
Benzodiazepine co‐prescribing is a trigger to deprescribe	“My first approach would be to reduce or eliminate the lorazepam because I think the polypharmacy would be my biggest concern off the bat.” (PCP 5, moderate‐risk)
Prioritizing mental health and social support prior to deprescribing.	“I don't think at the first visit I would lower anything, because I'm worried that he's going to make up the difference by getting it from the streets and he's going to see it as punishing him. I would ask if I'm allowed to speak to any family members and get them involved. I would get him plugged in with therapy” (PCP 11, high‐risk)

### 3.2 Approaches for Patients Open to Deprescribing

When asked about goals for deprescribing, for the low‐risk case, half of the PCPs wanted to reduce the dose, half did not have a specified goal, and none planned to taper the patient off completely. For the moderate‐risk patient, most specified the goal would be to reduce the dose, a few to taper off completely, and a few did not have a specified goal. For the high‐risk patient, PCPs were divided in their clinical reasoning as less than half did not have a specific taper goal, half had a goal of reducing the dose, and one to taper off completely (Figure S1B).

When asked about how they would approach a taper if the patient was open to deprescribing, PCPs often endorsed a patient‐directed deprescribing strategy for the low‐ and moderate‐risk cases (Table 3). One PCP described their approach to partnering around deprescribing: “My goal would be that we develop kind of a healthy partnership and conversation over the idea of tapering opioids so that it isn't seen as confrontational, but as I'm working with you and share your goal” PCPs did specify that deprescribing goals would be to prioritize patient function and for gradual dose reductions. One PCP described the balance between function and deprescribing as “If he tells me 3 months into the taper he's off the oxycodone but doesn't go for walks anymore; that's a huge problem.”

**TABLE 3:** Clinician approaches for patients willing to deprescribe.

Theme	Exemplary quotes
Patient‐directed tapering in low‐ and moderate‐risk cases.	“My goal would be that we develop kind of a healthy partnership and conversation over the idea of tapering opioids so that it isn't seen as confrontational, but as I'm working with you and share your goal.” (PCP 18, low‐risk).
Prioritize patient function	“I think that has to do with function…If he tells me 3 months into the taper he's off the oxycodone but doesn't go for walks anymore; that's a huge problem.” (PCP 8, low‐risk)
Emphasis on a gradual dose reduction	“The goal may not be to get off narcotics and may just be to lower the dose but I view that as a positive thing. But I would do it slowly and in small steps.” (PCP 6, moderate‐risk) “I would probably be looking at a 15% to 20% reduction for months… I would probably consider going down on his long acting by a little bit, and then we would see how he tolerates that” (PCP 14, high‐risk)
Treat with alternative opioid agonists	“Offer tapering down versus transition to [buprenorphine] and if he's not open to either of those, then it just seems to taper off of opioids because he sounds like he's going to continue to buy them on the street.” (PCP 7, high‐risk)
Consult with a pharmacist or specialist to assist with taper for high‐risk cases.	“If he was open for tapering and I was doing it, I would probably ask our pharmacists what is the best thing to do.” (PCP 6, high‐risk). “I don't have a ton of experience initiating [buprenorphine]. I've tried [buprenorphine/naloxone] for chronic pain more than for opioid use disorder, and so I would probably consult with [an addiction specialist].” (PCP 12, high‐risk)

In contrast, PCPs expressed less flexibility for the high‐risk case and those who would deprescribe would implement a mandatory structured taper. For example, one PCP stated that they would “Offer tapering down versus transition to [buprenorphine] and if he's not open to either of those, then it just seems to taper off of opioids because he sounds like he's going to continue to buy them on the street”. Some PCP advised treatment with an alternative opioid agonist to treat suspected OUD. Most PCPs would consult with a pharmacist or addiction specialist to assist with developing a deprescribing plan. Several PCPs also expressed concern that reducing opioids may predispose the patient to use illicit opioids, further increasing their risks.

### 3.3 Approaches for Patients Not Open to Deprescribing

For risk mitigation, naloxone was suggested by fewer than one‐third of PCPs across cases. For the high‐risk patient, approximately half of PCPs mentioned prescribing MOUD (i.e., buprenorphine or methadone)—which was a strategy not discussed in the low and moderate risk cases (Figure S1). PCPs offered a variety of risk mitigation strategies for patients who were not open to deprescribing (Table 4). For the low‐ and moderate risk cases, all PCPs planned to revisit or initiate deprescribing at a future follow‐up visit. One PCP described their plan approach as “I would tell her that I respect her choice not to talk about it right now… but will bring her back in three months and see how she's doing. And then by the second or third, I would really push for de‐escalating the dose”. PCPs reported they would focus on educating patients on the risks and side effects of opioids and reducing other medication related risks, for example by deprescribing benzodiazepines or removing the acetaminophen co‐formulated with oxycodone in the moderate risk case. One PCP emphasized framing conversations around education, stating “Education is probably the most important thing. Once they're aware of why I'm doing this, it's not taking away that pain medication. But to avoid side effects”. Another theme endorsed by PCPs was to maximize non‐opioid and non‐pharmacologic alternatives to treat pain.

**TABLE 4:** Risk mitigation strategies if patient was not open to deprescribing.

Theme	Exemplary quotes
Revisit deprescribing at future follow up visits in low‐ and moderate‐risk cases.	“We're going to touch base on this each time you're in for an appointment. It's to make sure that you remain informed and that if you change your mind, we have the opportunity to do something about it.” (PCP 14, low‐risk) “I would tell her that I respect her choice not to talk about it right now … but will bring her back in 3 months and see how she's doing. And then by the second or third, I would really push for de‐escalating the dose.” (PCP 18, moderate‐risk)
Educate patients on side effects and risks	“Education is probably the most important thing. Once they're aware of why I'm doing this, it's not taking away that pain medication.…But to avoid side effects.” (PCP 16, low‐risk)
Reduce other medications‐related risks.	“I will work with the lorazepam, use it maybe once a day or 0.25 mg twice a day and see how her anxiety feels with a lower dose.” (PCP 4, moderate‐risk). “I would probably switch her to oxycodone from oxycodone to acetaminophen …She's at risk of having acetaminophen overdose if it goes wrong.” (PCP 8, moderate‐risk)
Maximize non‐opioid and non‐pharmacologic treatment for pain.	“I would make sure that we tried other modalities that might help with their pain. So she'd go back to physical therapy. Maybe we reassess if she's a candidate for a knee replacement…Any sort of psychological counseling.” (PCP 2, moderate‐risk)
Addressing mental health concerns	“I will start with saying, why don't you talk to the psychiatrist and therapist and see if we can have your depression under control before tapering?” (PCP 16, high‐risk)
Pharmacologic treatment with naloxone or MOUD in high‐risk case.	“Make sure he has naloxone and anyone who lives with knows about it and how to use it.” (PCP 14, high‐risk) “Have a frank conversation about what's going on and then talk about treatments for that, whether it's Suboxone or methadone.” (PCP 12, high‐risk)
Stricter medication monitoring and/or tapering in high‐risk case	“If he is non‐adherent to the pain management agreement then I would still be doing the taper. This is the agreement and if you're not able to adhere to it, I'm not comfortable prescribing because I don't think it will be safe for you in the long term.” (PCP 14, high‐risk) “I would say that the presence of fentanyl in the urine is something that I cannot ignore…we are going to have to reduce the doses of medications.” (PCP 7, high‐risk)

For the high‐risk case, many PCPs would arrange acute mental health support via psychiatry or social work while some also identified referral to a pharmacist or addiction specialist for support. Many PCPs expressed a need for stricter oversight of patients due to safety concerns, including more frequent monitoring for patient adherence to subsequent prescriptions and either immediately tapering or enforcing taper if patients deviated from monitoring plans. For example, one PCP described an initial monitoring plan followed by mandated tapering “If he is non‐adherent to the pain management agreement then I would still be doing the taper. This is the agreement and if you're not able to adhere to it, I'm not comfortable prescribing because I don't think it will be safe for you in the long term”.

## 4 Discussion

In this qualitative case‐based study, we found that PCPs personalized their recommendations on reducing opioid use for older adults based on perceived risk of opioid‐related harms and that PCPs emphasized building partnership with patients. However, approaches to initiating conversations on opioid use, on how to deprescribe, and on how to mitigate opioid risks varied. PCPs generally tailored the urgency of deprescribing to their perception of patient risk for harms, advising patient‐led strategies for lower risk cases and more directive approaches for higher risk cases. PCPs endorsed discomfort with management decisions for the high‐risk case of a patient with signs of opioid use disorder, and were divided on whether to prioritize the reduction of opioid use or the management of mental health concerns.

These findings build on a prior study, which interviewed the same PCPs along with a cohort of older adults prescribed opioids for chronic pain, and which identified substantial disagreement between older adults and PCPs on the perceived risks of opioids and reasons to initiate deprescribing conversations [8]. The current study aimed to understand clinical approaches and decision‐making on deprescribing of the same PCPs. The Centers for Disease Control (CDC) guidelines recommend naloxone prescriptions in patients with MME > 50 [20]; however, in this study a low proportion of PCPs suggested naloxone co‐prescription or MOUD in the high‐risk case. This is consistent with the prior study which found PCPs caring for older adults were more concerned about adverse drug events than risks of overdose or OUD. Our findings are aligned with a prior survey which found that PCPs at Kaiser Permanente Washington frequently lacked confidence in engaging patients in opioid deprescribing or developing deprescribing plans [21]. Prior qualitative studies of Australian clinicians identified key barriers to opioid deprescribing such as varied prescribing practices and patient challenges, concluding similarly to our study that additional clinical guidance is needed to support clinicians [16, 22]. Prior US studies have investigated clinician perspectives on long‐term opioid treatment, finding a similar focus on developing trust and focusing on function, but these studies have not focused on specific issues encountered in the care of older adults [23, 24, 25].

The majority of clinicians expressed less confidence in the management of the case of an older adult with signs of OUD. Participants expressed both a need for support from other health care team members and a lack of comfort in managing OUD, despite the majority of PCPs being X‐waivered to prescribe MOUD. This finding is consistent with an identified lack of experience in treating OUD in primary care, and in particular with comfort with MOUD [26, 27, 28]. While legal barriers to treating OUD, such as the x‐waiver, have been removed, these findings serve as an important reminder that the ability to prescribe MOUD is only one component of safely managing high‐risk prescription opioid use and misuse.

The momentum for improving conversations on opioid prescribing is high – the 2022 CDC clinical practice guideline recommendation that prescribers discuss the benefits and harms of long‐term opioid therapy with patients every 3 months and 2023 Medicare guidance included a new monthly bundled payment code for managing chronic pain [19, 29]. Despite the perceived importance of discussing ongoing opioid use with older adults across the cases, our findings of variable practice patterns and discomfort with high‐risk situations indicate that PCPs require additional support and guidance tailored to patient risks. Educational tools, such as clinical frameworks or conversation aids, are needed to equip clinicians with skills to tailor conversations on long‐term opioid use, identify high‐risk features, and initiate deprescribing or other risk mitigation strategies when appropriate. Importantly, frameworks need to be developed explicitly for the population of older adults and adults with disability who face different risks and often fewer alternative chronic pain treatment options than younger populations. To date, CDC guidelines recommend “additional caution and increased monitoring” for older adults but practical guidance on this monitoring is limited to medication review, falls screening, and preventing constipation. One path forward may be developing conversation aids, which have been successful in supporting shared‐decision making between patients and older adults for other decisions such as ending cancer screening, advance care planning, and selecting among treatment options [30].

This study has limitations. The study included hospital‐based and community‐based clinicians in a single health system and may not be generalizable to other sites. Not all PCPs completed the case discussions due to time constraints. As cases were hypothetical, responses may be subject to social desirability, question order bias, or other response biases and may not reflect actual practice patterns. The updated CDC guideline for prescribing of opioids was released in November 2022 during the study period, which may have impacted responses.

In conclusion, this qualitative case‐based study of PCP approaches to managing opioids for older adults found that PCPs were less confident in their approaches to initiating deprescribing conversations, implementing a taper, and risk mitigation in patients at higher risk of opioid‐related harms and often expressed wanting additional support and guidance. PCPs may benefit from additional training and pragmatic tools to equip them with the evidence and appropriate risk mitigation strategies to implement successful conversations on opioid prescribing and deprescribing with older adults.

## Author Contributions

Ms. Wang has full access to all the data in the study and takes responsibility for the integrity of the data and the accuracy of the data analysis. Concept and design: Anderson, Lindenberg, Herzig, Schonberg. Acquisition, analysis, or interpretation of data: All authors drafting of the manuscript: Wang, Lindenberg. Critical review of the manuscript for important intellectual content: All authors statistical analysis: Anderson, Schonberg obtained funding: Anderson.

Administrative, technical, or material support: Anderson, Schonberg supervision: Schonberg, Anderson.

## Funding

Funded by the National Institute on Aging (R24AG064025). Dr. Anderson was additionally supported by grant K76AG074878 from the National Institute on Aging. Dr. Herzig was additionally supported by grant R01HS026215 from the Agency for Healthcare Research and Quality. Dr. Schonberg was additionally supported by grant K24AG071906 from the National Institute on Aging.

## Disclosure

The funders had no role in the design and conduct of the study; collection, management, analysis, and interpretation of the data; preparation, review, or approval of the manuscript; and decision to submit the manuscript for publication.

## Conflicts of Interest

Dr. Anderson reported receiving grants from the American Heart Association and Veterans Health Administration, and receiving personal fees from the American Medical Association outside the submitted work. Dr. Herzig reported receiving personal fees from DynaMed outside the submitted work. No other disclosures were reported.

## Supporting information


**Table S1:** Participant interview guide.
**Table S2:** Clinical cases completed by participant.
**Figure S1:** Opioid deprescribing decision‐making, by clinical case.

## References

[1] D. Dowell , T. M. Haegerich , and R. Chou , “CDC Guideline for Prescribing Opioids for Chronic Pain—United States, 2016,” Journal of the American Medical Association 315, no. 15 (2016): 1624–1645, 10.1001/jama.2016.1464. 26977696 PMC6390846

[2] A. Agnoli , G. Xing , D. J. Tancredi , E. Magnan , A. Jerant , and J. J. Fenton , “Association of Dose Tapering With Overdose or Mental Health Crisis Among Patients Prescribed Long‐Term Opioids,” Journal of the American Medical Association 326, no. 5 (2021): 411–419, 10.1001/jama.2021.11013. 34342618 PMC8335575

[3] T. L. Mark and W. Parish , “Opioid Medication Discontinuation and Risk of Adverse Opioid‐Related Health Care Events,” Journal of Substance Abuse Treatment 103 (2019): 58–63, 10.1016/j.jsat.2019.05.001. 31079950

[4] J. J. Fenton , A. L. Agnoli , G. Xing , et al., “Trends and Rapidity of Dose Tapering Among Patients Prescribed Long‐Term Opioid Therapy, 2008–2017,” JAMA Network Open 2, no. 11 (2019): e1916271, 10.1001/jamanetworkopen.2019.16271. 31730189 PMC6902834

[5] A. S. B. Bohnert , G. P. Guy , and J. L. Losby , “Opioid Prescribing in the United States Before and After the Centers for Disease Control and Prevention's 2016 Opioid Guideline,” Annals of Internal Medicine 169, no. 6 (2018): 367–375, 10.7326/m18-1243. 30167651 PMC6176709

[6] American Geriatrics Society , “Pharmacological Management of Persistent Pain in Older Persons,” Journal of the American Geriatrics Society 57, no. 8 (2009): 1331–1346, 10.1111/j.1532-5415.2009.02376.x. 19573219

[7] M. Hamilton , D. Gnjidic , C. W. Christine Lin , et al., “Opioid Deprescribing: Qualitative Perspectives From Those With Chronic Non‐Cancer Pain,” Research in Social & Administrative Pharmacy 18, no. 12 (2022): 4083–4091, 10.1016/j.sapharm.2022.07.043. 35963766

[8] T. S. Anderson , B. X. Wang , J. H. Lindenberg , S. J. Herzig , D. M. Berens , and M. A. Schonberg , “Older Adult and Primary Care Practitioner Perspectives on Using, Prescribing, and Deprescribing Opioids for Chronic Pain,” JAMA Network Open 7, no. 3 (2024): e241342, 10.1001/jamanetworkopen.2024.1342. 38446478 PMC10918495

[9] A. Dufort and Z. Samaan , “Problematic Opioid Use Among Older Adults: Epidemiology, Adverse Outcomes and Treatment Considerations,” Drugs & Aging 38, no. 12 (2021): 1043–1053, 10.1007/s40266-021-00893-z. 34490542 PMC8421190

[10] C. Shoff , T. C. Yang , and B. A. Shaw , “Trends in Opioid Use Disorder Among Older Adults: Analyzing Medicare Data, 2013–2018,” American Journal of Preventative Medicine 60, no. 6 (2021): 850–855, 10.1016/j.amepre.2021.01.010. PMC8154702 33812694

[11] I. A. Scott , S. N. Hilmer , E. Reeve , et al., “Reducing Inappropriate Polypharmacy: The Process of Deprescribing,” JAMA Internal Medicine 175, no. 5 (2015): 827–834, 10.1001/jamainternmed.2015.0324. 25798731

[12] S. Mathieson , C. G. Maher , G. E. Ferreira , et al., “Deprescribing Opioids in Chronic Non‐Cancer Pain: Systematic Review of Randomised Trials,” Drugs 80, no. 15 (2020): 1563–1576, 10.1007/s40265-020-01368-y. 32737739

[13] C. Eccleston , E. Fisher , K. H. Thomas , et al., “Interventions for the Reduction of Prescribed Opioid Use in Chronic Non‐Cancer Pain,” Cochrane Database of Systematic Reviews 11, no. 11 (2017): CD010323, 10.1002/14651858.cd010323.pub3. 29130474 PMC6486018

[14] J. D. Niznik , B. J. Collins , L. T. Armistead , et al., “Pharmacist Interventions to Deprescribe Opioids and Benzodiazepines in Older Adults: A Rapid Review,” Research in Social & Administrative Pharmacy 18, no. 6 (2021): 2913–2921, 10.1016/j.sapharm.2021.07.012. 34281786 PMC8836277

[15] J. D. Niznik , S. P. Ferreri , L. T. Armistead , et al., “Primary‐Care Prescribers' Perspectives on Deprescribing Opioids and Benzodiazepines in Older Adults,” Drugs & Aging 39, no. 9 (2022): 739–748, 10.1007/s40266-022-00967-6. 35896779 PMC9330848

[16] M. Hamilton , S. Mathieson , D. Gnjidic , et al., “Barriers, Facilitators, and Resources to Opioid Deprescribing in Primary Care: Experiences of General Practitioners in Australia,” Pain 163, no. 4 (2021): e518–e526, 10.1097/j.pain.0000000000002340. 33990105

[17] A. Tong , P. Sainsbury , and J. Craig , “Consolidated Criteria for Reporting Qualitative Research (COREQ): A 32‐Item Checklist for Interviews and Focus Groups,” International Journal for Quality in Health Care 19, no. 6 (2007): 349–357, 10.1093/intqhc/mzm042. 17872937

[18] Stakeholder Engagement Council—US Deprescribing Research Network , “US Deprescribing Research Network,” (2025), https://deprescribingresearch.org/stakeholder‐engagement‐council/.

[19] H. F. Hsieh and S. E. Shannon , “Three Approaches to Qualitative Content Analysis,” Qualitative Health Research 15, no. 9 (2005): 1277–1288, 10.1177/1049732305276687. 16204405

[20] D. Dowell , K. Ragan , C. Jones , G. Baldwin , and R. Chou , “CDC Clinical Practice Guideline for Prescribing Opioids for Pain,” MMWR ‐ Recommendations and Reports 71, no. 3 (2022): 1–95, 10.15585/mmwr.rr7103a1. PMC9639433 36327391

[21] S. L. Gray , R. Fornaro , J. P. Turner , et al., “Provider Knowledge, Beliefs, and Self‐Efficacy to Deprescribe Opioids and Sedative‐Hypnotics,” Journal of the American Geriatrics Society 71, no. 5 (2022): 1580–1586, 10.1111/jgs.18202. 36546768

[22] A. V. Langford , D. Gnjidic , C. W. C. Lin , et al., “Challenges of Opioid Deprescribing and Factors to Be Considered in the Development of Opioid Deprescribing Guidelines: A Qualitative Analysis,” BMJ Quality and Safety 30, no. 2 (2020): 133–140, 10.1136/bmjqs-2020-010881. 32220937

[23] A. L. Nevedal , C. Timko , M. C. Lor , and K. J. Hoggatt , “Patient and Provider Perspectives on Benefits and Harms of Continuing, Tapering, and Discontinuing Long‐Term Opioid Therapy,” Journal of General Internal Medicine 38, no. 8 (2022): 1802–1811, 10.1007/s11606-022-07880-z. 36376623 PMC9663196

[24] L. G. Militello , R. W. Hurley , R. L. Cook , et al., “Primary Care Clinicians' Beliefs and Strategies for Managing Chronic Pain in an Era of a National Opioid Epidemic,” Journal of General Internal Medicine 35, no. 12 (2020): 3542–3548, 10.1007/s11606-020-06178-2. 32909230 PMC7728906

[25] J. J. Wyse , L. Ganzini , S. K. Dobscha , E. E. Krebs , and B. J. Morasco , “Setting Expectations, Following Orders, Safety, and Standardization: Clinicians' Strategies to Guide Difficult Conversations About Opioid Prescribing,” Journal of General Internal Medicine 34, no. 7 (2019): 1200–1206, 10.1007/s11606-019-04983-y. 31011964 PMC6614300

[26] A. A. Bergman , R. S. Oberman , S. L. Taylor , B. Kranke , and E. T. Chang , “Prescribing and Acceptance of Medications for Opioid Use Disorder in VA Primary Care: Veteran and Provider Perspectives,” Journal of General Internal Medicine 39, no. 9 (2024): 1690–1697, 10.1007/s11606-024-08703-z. 38587730 PMC11254870

[27] E. J. Austin , J. Chen , E. Soyer , et al., “Optimizing Patient Engagement in Treatment for Opioid Use Disorder: Primary Care Team Perspectives on Influencing Factors,” Journal of General Internal Medicine 39, no. 16 (2024): 3196–3204, 10.1007/s11606-024-08963-9. 39073482 PMC11618257

[28] S. N. Kapadia , J. L. Griffin , J. Waldman , N. R. Ziebarth , B. R. Schackman , and C. N. Behrends , “A Harm Reduction Approach to Treating Opioid Use Disorder in an Independent Primary Care Practice: A Qualitative Study,” Journal of General Internal Medicine 36, no. 7 (2021): 1898–1905, 10.1007/s11606-020-06409-6. 33469774 PMC7815286

[29] “Calendar Year (CY) 2023 Medicare Physician Fee Schedule Final Rule | CMS,” (2022), https://www.cms.gov/newsroom/fact‐sheets/calendar‐year‐cy‐2023‐medicare‐physician‐fee‐schedule‐final‐rule.

[30] D. Stacey , F. Légaré , K. Lewis , et al., “Decision Aids for People Facing Health Treatment or Screening Decisions,” Cochrane Database of Systematic Reviews 4, no. 4 (2017): CD001431, 10.1002/14651858.CD001431.pub5. 28402085 PMC6478132

