# Supplementary material for: Primary Care Practitioners' Approaches to Deprescribing Opioids for Older Adults With Chronic Pain: A Qualitative Analysis
Source: J Am Geriatr Soc. 2026 Apr 8;74(6):1638–46. doi: 10.1111/jgs.70438 (PMC13266437; doi:10.1111/jgs.70438)
Supplement: Supplementary file 1 — Table S1: Participant interview guide. Table S2: Clinical cases completed by participant. Figure S1: Opioid deprescribing decision‐making, by clinical case. [file JGS-74-1638-s001.pdf]

Supplementary Table S1: Participant Interview Guide

Refer to the Text Box that contains the clinical cases. The following questions were asked for each case in sequential order.

1. Would you talk with him/her about reducing him use of opioids? Explain your thought process and what factors you would consider when deciding whether or not to talk with him/her.
    a. If you would talk with him/her, how would you approach that conversation?
    b. If you would not talk with him/her, would you take any other steps to mitigate risk?
2. If the patient was open to tapering his opioids, how would you begin? If you aren't sure, what resources would you use to determine this?
    a. What would be your goal?
    b. How would you assess progress?
3. If the patient was not open to tapering her opioids, what would you do? Would you take any additional steps to mitigate risks?

Supplementary Table S2: Clinical Cases Completed by Participant

Record ID#	Low Risk Case	Moderate Risk Case	High Risk Case
**1**		X	
**2**		X	X
**3**	X	X	X
**4**		X	X
**5**		X	X
**6**	X	X	X
**7**		X	X
**8**	X	X	X
**9**	X	X	X
**10**	X	X	
**11**	X	X	X
**12**	X	X	X
**13**	X	X	X
**14**	X	X	X
**15**	X	X	X
**16**	X	X	X
**17**	X	X	X
**18**	X	X	X

Supplementary Figure S1: Opioid Deprescribing Decision-making, by Clinical Case

A) Initiation of Deprescribing Conversation

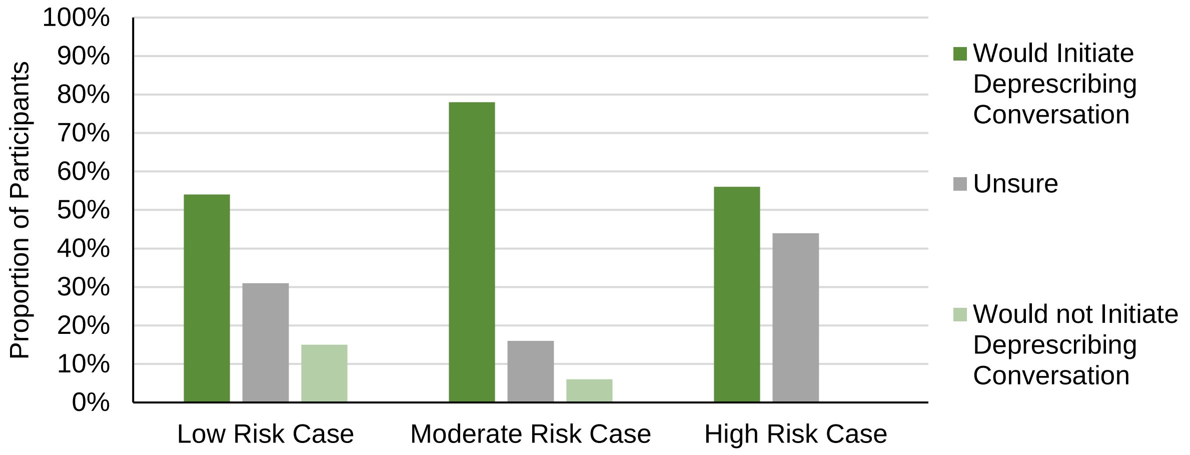

B) Tapering Goal if Patient was Open to Tapering

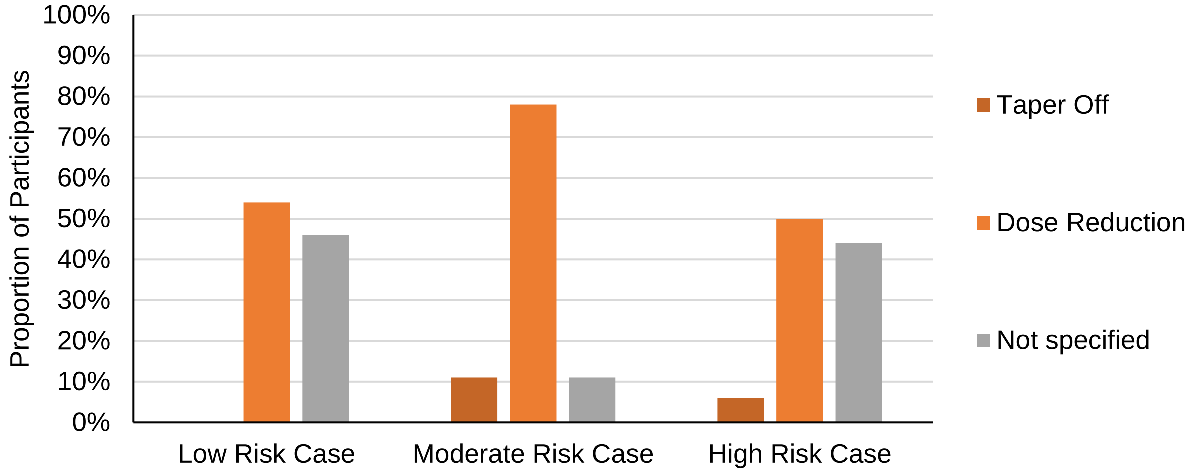

C) Use of Pharmacotherapy for Risk Mitigation

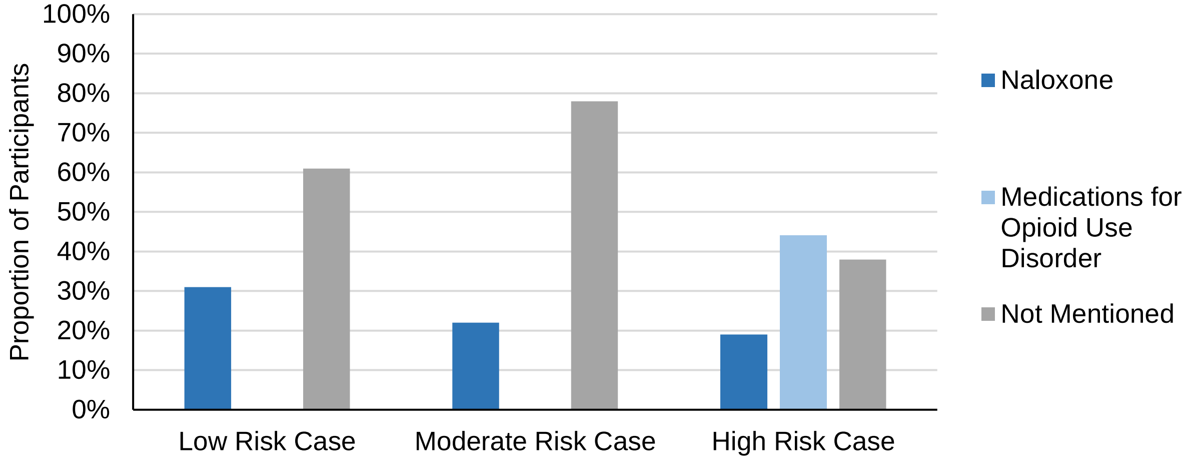

*Footnote: N = 13 for low-risk case, N = 18 for moderate-risk case, and N = 16 for high-risk case. For panel A, P-value for Fisher's exact test comparing responses across cases 0.11. For Panel B, P-value for Fisher's exact test

comparing responses across cases 0.13. For panel C, P-value for Fisher's exact test comparing uses of naloxone across cases 0.83 and use of MOUD across cases <.001.
